# Multi-Component Comparative Pharmacokinetics in Rats After Oral Administration of *Fructus aurantii* Extract, Naringin, Neohesperidin, and Naringin-Neohesperidin

**DOI:** 10.3389/fphar.2020.00933

**Published:** 2020-06-19

**Authors:** Jinbin Yuan, Feiting Wei, Xizhen Luo, Min Zhang, Rifa Qiao, Minyong Zhong, Haifang Chen, Wuliang Yang

**Affiliations:** ^1^Key Laboratory of Modern Preparation of TCM, Ministry of Education, Jiangxi University of Traditional Chinese Medicine, Nanchang, China; ^2^Nanchang Key Laboratory of Quality Control and Safety Evaluation of Traditional Chinese Medicine, Nanchang Institute for Food and Drug Control, Nanchang, China

**Keywords:** Citrus × aurantium L., *Fructus aurantii*, naringin, neohesperidin, pharmacokinetics, ultra-high performance liquid chromatography-tandem mass spectrometry

## Abstract

Citrus × aurantium L., Chinese name: *Fructus Aurantii* (FA) has been largely used as Qi-invigorating herb in China for centuries. The main components (meranzin hydrate, naringin, neohesperidin, meranzin, nobiletin) have good physiological activity with relatively high abundance in FA. Few multi-component comparative pharmacokinetics are simultaneously accessible for the flavone glycosides, polymethoxy flavones, and coumarins in FA. In this work, a reliable and rapid ultra-high performance liquid chromatography-tandem mass spectrometry (UPLC-MS/MS) method was established and validated to determine the five ingredients in the SD rat plasma, and further applied to the pharmacokinetic studies after oral administration of monomer, drugs in compatibility, and FA extract. After hydrolysis with *β*-glucuronidase and sulfatase, the concentration of naringin and neohesperidin in rat plasma were expressed respectively by the total concentration of naringenin and hesperitin which was determined by UPLC-MS/MS. Double-peak phenomenon was observed for naringin and neohesperidin, which may be due to the enterohepatic circulation or multiple site absorption of the two flavone glycosides. Meranzin hydrate and meranzin (coumarins) were absorbed rapidly (T_max,_ about 1.0 h) but eliminated slowly (t_1/2z_ exceeds 6.5 h). Nobiletin, a typical polymethoxy flavone, was also rapidly absorbed according to T_max_ and AUC_(0-t)_. DAS 3.1 software suggests the pharmacokinetic profiles of the five components in rats be depicted as a two-compartment pharmacokinetic model. There were significant differences in pharmacokinetic parameters for naringenin and hesperetin between the compatibility, FA extract group *vs* monomer group: ① remarkable increases in the values of AUC_(0–∞)_, AUC_(0–t)_ and C_max_; ② obvious decrease of CL_Z/F_; and ③ longer t_max_ and t_1/2z_. The results suggest that compatibility can promote mutual absorption and affect the elimination behaviors.

## Introduction

*Fructus aurantii* (FA), also called Zhiqiao in Chinese, refers to the dried unripe fruit of *Citrus* × *aurantium* L. or its cultivar (Rutaceae). FA is a very important Qi-invigorating herb in China for thousands of years, and it vigorously modulates the motion of Qi and fortifies the spleen and stomach (Committee of National Pharmacopoeia of RP China, 2015). Modern pharmacological researches have established that FA possesses astoundingly biomedical properties, such as prokinetic effect (Qiu et al., 2011; Zhou et al., 2018), antidepression activity (Wu et al., 2015), anti-inflammation function (Parhiz et al., 2015), anticarcinogenic activity (Park et al., 2014), antihepatotoxicity (Renugadevi and Prabu, 2010), antihypersensitivity (Kobayashi et al., 2015), and hypoglycemic and hypolipidemic effects (Jia et al., 2015). It is well known that the primary chemical compositions in FA are flavonoids, alkaloids, volatile oils, and coumarins (Li et al., 2016). In which the main bioactive compounds are considered to be naringin, neohesperidin, meranzin hydrate (MH), meranzin, Naringenin, hesperetin nobiletin, tangeretin, and auraptene (Qiu et al., 2011; Yuan et al., 2012; Li et al., 2016; Wang et al., 2018; Zhang et al., 2018; Chen et al., 2019).

The therapeutic effects of a drug and its components are closely related to their pharmacokinetic characteristics. The pharmacokinetics of naringin and/or neohesperidin have been extensively studied in rats (Felgines et al., 2000; Fang et al., 2006; Li et al., 2010; Lee et al., 2017; Zeng et al., 2019), dogs (Mata-Bilbao et al., 2007), rabbits (Hsiu et al., 2002), and human beings (Ishii et al., 1996; Ishii et al., 1997; Bai et al., 2020). Multi-component pharmacokinetics can also be accessible for several flavones in FA (Tong et al., 2012; Wang et al., 2018; Zhang et al., 2018). Very recently, the comparative pharmacokinetics of naringin and neohesperidin between different model rats (Xu et al., 2019) and different herbs (Li et al., 2019) have been reported. As a whole, the above pharmacokinetic evaluation mainly focused on pure monomers (mainly flavones) (Ishii et al., 1996; Ishii et al., 1997; Felgines et al., 2000; Hsiu et al., 2002; Fang et al., 2006; Mata-Bilbao et al., 2007; Li et al., 2010; Lee et al., 2017), the extract of a single herb (Tong et al., 2012; Wang et al., 2018; Zhang et al., 2018), or the study of the pharmacokinetic interaction between two or more herbs (Li et al., 2019; Xu et al., 2019). Few pharmacokinetic data can be accessible for the other compounds, such as MH, meranzin, and nobiletin.

Our previous study indicated that the components (MH, meranzin, and nobiletin) have good physiological activity with relatively high abundance in FA besides naringin and neohesperidin. We also found that the drug quality is closely related to the content proportion of naringin *vs* neohesperidin in FA, which can partly elucidate the geo-authentic origin of the medicinal plant (Chen et al., 2019). So, it is interesting to investigate the pharmacokinetic differences of naringin and neohesperidin between the different forms (monomer, compatibility, and extract).

Some literatures indicated that naringin and neohesperidin transiently exist in the plasma, and the glucuronides/sulfates of their aglycones (naringenin and hesperetin) were the main circulating metabolites (Felgines et al., 2000; Fang et al., 2006; Xu et al., 2019). Free naringenin and hesperetin can be easily obtained from the naringenin-glucronaide and hesperetin-glucronaide in plasma by hydrolysis with *β*-glucuronidase and sulfatase. In addition, naringenin and hesperetin are relatively low content in FA. So, the concentration of the absorbed naringin and neohesperidin can be detected in the corresponding aglycone form (naringenin and hesperetin).

Hence, a rapid and sensitive ultra-high performance liquid chromatography-tandem mass spectrometry (UPLC-MS/MS) method was developed and validated for the simultaneous determination of the main components (naringin, neohesperidin, MH, meranzin, and nobiletin) in rat plasma, and used for the pharmacokinetic study of the compounds in different forms: monomer, compatibility, and extract.

## Materials and Methods

### Crude Drugs

*Fructus aurantii*, the dry immature fruit of *Citrus × aurantium* L., were provided by Jiangxi Zhihui Chinese Medicinal Materianls Co. Ltd. The coordinates of plant picking is 115.226067 East longitude and 28.030638 North latitude (Wucheng Town, Zhangshu, Jiangxi, China). The crude drugs were identified as *Citrus junos* Sieb. ex Tanaka by Professor Wuliang Yang from Jiangxi University of Traditional Chinese Medicine (JXUTCM), China. Voucher specimens are preserved in the Herbarium of Pharmacognosy in JXUTCM.

### Chemicals and Reagents

Standards including neohesperidin, naringin, meranzin hydrate, naringenin, hesperetin, meranzin, and nobiletin were purchased from Chengdu Desite Biotech Co., Ltd. (Chengdu, China), and their purities were not lower than 98% according to HPLC analysis. Quercetin (Internal standard, IS) (HPLC ≥ 98%) was purchased from Chengdu Mansite Pharmaceticcal Co., Ltd. (Chengdu, China). *β*-glucuronidase and sulfate esterase were purchased from Shanghai Yuanye Bio-Technology Co., Ltd. (Shanghai, China), Formic acid was purchased from Shanghai Aladdin Bio-Chem Technology Co. Ltd. (Shanghai, China). Chromatographic reagent Methanol (Merck, Germany) and acetonitrile (Tedia, USA) were used throughout. Deionized Water was purified by Milli-Q purification system (Millipore, MA, USA). All other chemical regents were of analytical grade.

### Apparatus and UPLC–MS/MS Conditions

The UPLC-MS/MS system consisting of Shimadzu Ultra-High Performance Liquid Chromatograph LC-30A and Triple Quadrupole Mass Spectrometer (LCMS-8050, SHIMADZU, Japan).

#### Liquid Chromatography

The chromatographic separation was achieved on an Agilent C_18_ column (2.1x100mm, 1.8μm, Agilent, United States) with temperature at 40°C; The mobile phase was composed of water (containing 0.1% formic acid) (solvent A) and Acetonitrile (solvent B). The gradient elution procedures (0.01–5 min, 30 to 50% B, 5–7min, 50% B, 7–7.5min, 50–30% B, 7.5–10.5min, 30% B); The flow rate was 0.3 ml/min and injection volume was 1.0 μl (Yuan et al., 2017).

#### Mass Spectrometry

The mass spectrometer was operated in negative mode for quercetin (IS), naringenin, and hesperetin, and positive mode for meranzin hydrate, meranzin, nobiletin. Quantification was obtained using multiple reaction monitoring (MRM) mode in the following positive/negative MS/MS scan segments. Segment I: 1.5–2.3min in positive mode, meranzin hydrate; Segment II: 2.2–4.5min in negative mode quercetin (IS), naringenin, and hesperetin; Segment III: 4.4–7.5min in positive mode for meranzin and nobiletin. The optimal MS parameters were as follows: Interface ESI, Nebulizing Gas Flow of 3.0 L/min; Heating Gas Flow of 10 L/min; Interface Temperature of 300°C; DL Temperature of 250°C; Heat Block Temperature of 400°C; Drying Gas Flow of 10 L/min; Nebulizing Gas of 3.0 L/min, Drying Gas F1 of 10.0 L/min, Conversion Dy: 10 kV, Detector Volt: 1.92 kV. MS/MS operating conditions were optimized by infusion of the standard solution (200 ng/ml) of each analyte and I.S. into the ESI source *via* a syringe pump. The optimal MRM parameters for these compounds are shown in **Table 1**.

**Table 1:** MRM detection parameters for the five components and IS.

Components	Precursor ion	Q_1_ (V)	Collision energy (eV)	Q_3_ (V)	Product ion
Meranzin hydrate	279.10	-13.00	-33.00	-25.0	131.10
Naringenin	271.10	13.00	18.00	14.00	151.05
Hesperetin	301.05	15.00	24.00	15.00	164.05
Meranzin	261.10	-12.00	-29.00	-26.0	131.10
Nobiletin	403.15	-18.00	-28.00	-27.00	373.10
Quercetin (IS)	301.10	14.00	22.00	13.00	151.00

### Animals

Male Sprague-Dawley (SD) rats [Certificate No. SCXK(Xiang) 2016-0002] weighing 200 ± 20 g were purchased from Hunan Silaike Laboratory Animal Ltd (Changsha, China). The animals were kept in a controlled breeding room with the following conditions: a temperature of 22 ± 2°C, a relative humidity of (65 ± 5)%, and a 12 h light- dark cycle (Yuan et al., 2017). The Experimental Animal Ethic Committee of JXUTCM approved all animal protocols. The animal experiments were carried out according to the European Community guidelines for the use of experimental animals.

### Sample Preparation

#### Preparation of FA Extract

The dried drug was extracted twice by refluxing with boiling Methanol (1:10, w/v) for 2 h, then the two filtrates were merged and evaporated to dryness under vacuum (Tong et al., 2012). The extract (14.56 g) was weighed and the extract yield was 29.14%. The obtained extract was stored at 4 °C until use. To count the dosage, the contents of the main compounds in the FA extract were quantitatively measured by HPLC (Zhang et al., 2018; Chen et al., 2019), and the contents of naringin, neohesperidin, meranzin hydrate, meranzin and nobiletin in the extract were 171.6, 167.5, 1.98, 0.94, and 1.70 mg/g raw herb, respectively (see supplementary material: **Figure S1**).

#### Preparation of Calibration Standards and Quality Control (QC) Samples

The stock solutions of the mixed standards were prepared in methanol with the concentrations of meranzin hydrate (194 µg/ml), naringenin (491 µg/ml), hesperetin (494 µg/ml), meranzin (209 µg/ml), nobiletin (528 µg/ml), and quercetin (IS) (303 µg/ml). All solutions were stored at 4 °C. The concentrations of each analyte in standard mixture solutions were as follows: 19.4 µg/ml for meranzin hydrate, 14.73 µg/ml for naringenin, 12.35 µg/ml for hesperetin, 4.18 µg/ml for meranzin, and 2.112 µg/ml for nobiletin. Then, the standard mixture solution was diluted to a series of concentration (1/2, 1/5, 1/10, 1/20, 1/50, 1/100, 1/250, 1/500, 1/1,000, and 1/2,000) as calibration curves. QC samples were prepared at three concentration levels containing meranzin hydrate (19.4, 388, and 19,400 ng/ml), naringenin (14.73, 294.6, and 14,730 ng/ml), hesperetin (12.35, 247, and 12,350 ng/ml), meranzin (4.18, 83.6, and 4,180 ng/ml), and nobiletin (2.112, 42.2, and 2,112 ng/ml). These samples were stored at −80°C until analysis.

#### Biological Sample Preparation

The 20 μl of *β*-glucuronidase and 15 μl of sulfatase were added into 50 μl of plasma sample. After vortexed for 30 s, the mixture was incubated at 37°C for 2 h in a water bath, then placed in ice water for 5 min to terminate the reaction (Xu et al., 2019). IS solution (20 µl, 5 μg/ml) and acetonitrile (1.0 ml) were added into the mixture and vortexed for 2 min. The mixture was centrifuged at 4,000 rpm at 4°C for 10 min, and the supernatant (850 µl) was transferred into a new eppendorf tube and evaporated to dryness. The residue was redissolved in 100 μl of 50% methanol, and vortexed for 3 min, then centrifuged at 14,000 rpm at 4°C for 10 min, finally the supernatant was used for UPLC-MS/MS analysis.

### Method Validation

This method was validated according to the current US FDA Bioanalytical Method Validation Guidance (Guidance for Industry: Bioanalytical Method Validation, 2001). The following parameters were determined: selectivity, linearity, LLOQ, LOD, precision, accuracy, extraction recovery, matrix effect, and stability.

#### Linearity Parameters

To assess the linearity ranges, a series of the mixed standard solutions (six concentration levels) were prepared in triplicate. Each calibration curve (y = ax + b) was established by plotting the peak area ratio of analyte to IS (y) against the concentrations (x) of the calibration solution with a least square linear regression. The limit of detection (LOD) and limit of quantification (LOQ) were determined when the peak height was three times and ten times the background noise, respectively (Yuan et al., 2017).

#### Specificity and Selectivity

Blank plasma samples or the samples spiked with the IS were detected for endogenous or IS interference (Zhang et al., 2018). Specificity and selectivity were investigated by comparing the chromatograms of five individual batches of blank plasma samples, the samples spiked with the 5 analytes and IS, and plasma samples at 0.75 h after oral administration of FA extracts.

#### Accuracy and Precision

The intra- and inter-day precisions and accuracies were calculated by an analysis of variance based on the replicate analysis of QC samples, and the work was accompanied by a standard calibration curve on each analytical run (Yuan et al., 2017). Five samples were measured for each concentration level on the optimal conditions five times within the same day for intra-day variance, and the three different days for inter-day variance. The analytic precision was denoted as the RSD (relative standard deviation), and the accuracy was considered the RE (relative error) of the measured average deviated from the nominal value. The precision and accuracy values (RSD%) were required to be within ±20% for LLOQ (lower limit of quantification) and within ±15% for other concentrations.

#### Extraction Recovery and Matrix Effect

The extraction recoveries of the compounds were assessed by comparing the mean peak areas from five plasma samples in the pre-extraction spiked with the analytes and the post-extraction at three QC levels. The matrix effects were evaluated by comparing the peak areas obtained from the spiked plasma matrix with the pure standard solutions at the same concentrations. There was no matrix effect if the ratio was between 85–115%.

#### Stability

The QC samples were assayed under several different conditions to evaluate the stability of the compounds in rat plasma. For the short-term stability, fresh plasma samples were kept at room temperature for 24 h before the sample preparation. After keeping the samples at room temperature for 24 h, post-preparation stability was tested. Freeze-thaw cycles (-20 °C/room temperature) stability were not treated at -20°C for freezing-room temperature melting three times (repeated freezing and thawing three times). In each freeze-thaw cycle, the samples were frozen and stored at -20 °C for 24 h, and subsequently thawed at room. To evaluate the long-term stability of QC plasma samples, following storage at -20 °C for one month. The stability data were acceptable when the bias was within ±15% of the actual value (Yuan et al., 2017).

### Pharmacokinetics

Rats were randomly divided into the following four groups (6 rats in each group): naringin (NA), neohesperidin (NHE), naringin-neohesperidin (NA-NHE), and FA extract group. The oral administration doses were selected according to the literatures (Tong et al., 2012; Yang et al., 2012; Committee of National Pharmacopoeia of RP China, 2015; Wang et al., 2018; Zhang et al., 2018; Chen et al., 2019), the contents of the five components in FA, and the preliminary experiments. The specific doses were as follows: FA extract (10.8 g/kg of the original medicinal material), 10 ml/kg; naringin and neohesperidin, 1.85 g/kg and 1.81 g/kg, respectively. The drug was suspended in 0.5% CMC-Na.

Blood samples (about 0.5 ml) were collected from the ocular vein using dried heparinized tubes from each six rats at 0, 0.083, 0.167, 0.25, 0.5, 0.75, 1, 1.5, 2.0, 4.0, 6.0, 8.0, 10.0, 12.0, 14.0, 24.0, and 30.0 h after dosing. The rats were intragastric administrated with 1.5 ml of water at the time points of 0.25, 0.5, 2, 4, and 6 h, respectively. The rats had free access to water during the experiment. The blood samples were then immediately centrifuged at 4,000 rpm for 10 min. The plasma was frozen and stored at −80°C until analysis.

The plasma concentration of the studied compounds was determined according to the daily calibration curve. The pharmacokinetic parameters, including C_max_ (the peak drug plasma concentration), T_max_ (the time to C_max_), AUC_(0-t)_ (the area under the plasma concentration-time curve from 0 to the last time point), AUC_(0−∞)_ (the area under the plasma concentration-time curve from 0 to time infinity), MRT_(0–t)_ (average residence time from 0 to the last time point), and t_1/2z_ (the elimination half-life) were fitted with the proprietary DAS 3.1 pharmacokinetic program (Chinese Pharmacological Society).

### Statistical Analysis

All statistical analyses were performed using the SPSS 21.0 software package from SPSS, Inc. (Chicago, IL, USA). *P values < 0.05, or **P values < 0.01 was considered statistically significant difference.

## Results

### UPLC-MS/MS Conditions

**Figure 1:**
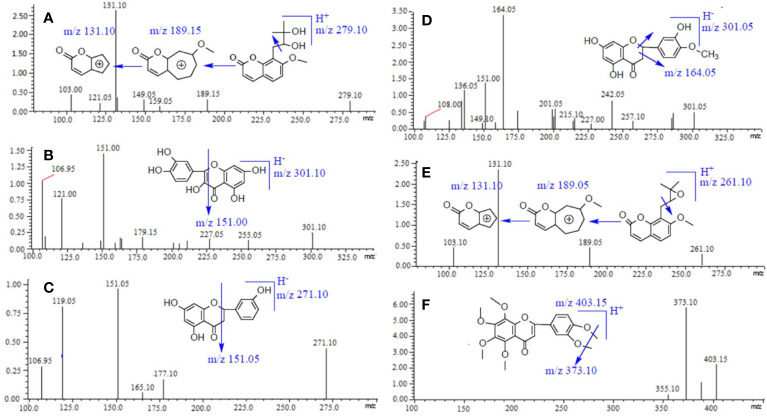
Chemical structures and product ion mass spectra of Meranzin hydrate **(A)**, Quercetin (IS) **(B)**, Naringenin **(C)**, Hesperidin **(D)**, Meranzin **(E)**, and Nobiletin **(F)**.

According to the direct full-scan ESI mass spectra, the ion intensities of naringenin, hesperetin, and quercetin (IS) was higher in negative mode, and the other three compounds (meranzin hydrate, meranzin, and nobiletin) got greater sensitivity in positive mode. So, the switch scan method (ESI +/-) was used for the corresponding compounds, and the product ion mass spectra of the six compounds are shown in **Figure 1**.

Based on previous work, chromatographic conditions such as the constituents and the gradient profiles of the mobile phase were further improved to adapt the separation and detection of six components in rat plasma. In addition, the optimum mobile phase was water (containing 0.1% formic acid) and acetonitrile.

Various sample preparation methods including protein precipitation with methanol and acetonitrile were investigated to obtain an efficient cleanup of the biological samples. In this work, acetonitrile was chosen as the protein precipitation agent with simplicity and efficiency.

### Specificity

**Figure 2:**
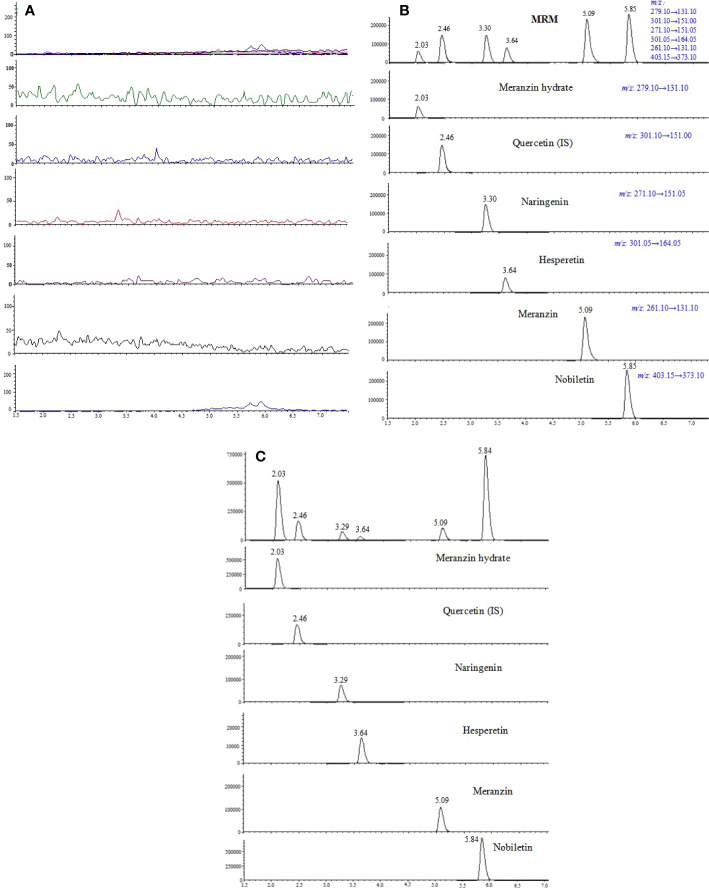
Representative multiple reaction monitoring (MRM) chromatograms of meranzin hydrate, naringenin, hesperetin, meranzin, nobiletin, and quercetin (IS) in rat plasma: **(A)** blank plasma, **(B)** blank plasma spiked with the 5 analytes and quercetin (IS), and **(C)** plasma sample collected at 0.75 h after oral administration of FA extract (10.8 g/kg) to rats.

Typical chromatograms of blank, spiked plasma and plasma samples are shown in **Figure 2**. No interference was found between endogenous compounds or xenobiotics. The developed method results in six single sharp peaks at the retention time of meranzin hydrate, quercetin (IS), naringenin, hesperetin, meranzin, and nobiletin were 2.03, 2.46, 3.29, 3.63, 5.09, and 5.84 min, respectively.

### Linearity Parameters

The linearity parameters of the five components in rat plasma using weighed (1/x^2^) least-squares linear regression analysis are summarized in **Table 2**. Within the investigated linear range, good linearity data was obtained with correlation coefficients >0.9941. The results indicate the proposed UPLC-MS/MS method has a wide linear range and high sensitivity.

**Table 2:** Linear parameters of the compounds in rat plasma.

Components	Calibration curves	Correlation coefficients	Range (ng/mL)	LLOQ (ng/mL)	LOD (ng/mL)
Meranzin hydrate	y = 0.001*x* + 0.0653	r^2^ = 0.9941	1.94 ~ 3880	1.94	0.59
Naringenin	y = 0.0021*x* + 0.0383	r^2^ = 0.9981	1.473 ~ 2946	1.473	0.45
Hesperetin	y = 0.0017*x* - 0.0031	r^2^ = 0.9994	1.235 ~ 2470	1.235	0.37
Meranzin	y = 0.0207*x* - 0.0033	r^2^ = 0.9998	0.418 ~ 836	0.418	0.13
Nobiletin	y = 0.0778*x* - 0.1077	r^2^ = 0.9961	0.2112 ~ 422.4	0.2112	0.06

### Precision and Accuracy

The precision and accuracy results are shown in **Table 3**, intra-day precision was less than 7.9%, intra-day accuracy was between -8.66 to 9.68%, and inter-day precision was less than 10% for the five compounds. Inter-day accuracies were in the range of -9.34–9.94% expressed by relative errors (RE%). The relative standard deviation and relative error values did not exceed ± 15% (**Table 3**), this indicate the accuracy and precision are acceptable.

**Table 3:** Intra-day and inter-day precisions and accuracies of the analytes from QC samples prepared in rat plasma (n=6).

Components	Spiked concentration (ng/mL)	Intra-day			Inter-day		
		Concentration measured (ng/mL)	Precision (RSD,%)	Accuracy (RE,%)	Concentration measured (ng/mL)	Precision (RSD,%)	Accuracy (RE,%)
Meranzin hydrate	3.88	3.94 ± 0.10	2.6	1.52	3.98 ± 0.24	6.0	2.65
	77.6	73.57 ± 5.77	7.8	5.19	71.38 ± 4.24	6.0	-8.01
	3880	3557.39 ± 20.27	0.6	8.31	3517.64 ± 56.07	1.6	-9.34
Naringenin	2.946	2.92 ± 0.16	5.6	-0.98	2.97 ± 0.21	7.2	0.69
	58.92	64.03 ± 3.83	6.0	8.68	63.79 ± 4.18	6.6	8.27
	2946	2981.99 ± 39.27	1.3	1.22	2969.13 ± 58.10	2.0	0.78
Hesperetin	2.47	2.62 ± 0.06	2.3	6.02	2.56 ± 0.15	5.8	3.50
	49.4	51.38 ± 3.35	6.5	4.01	50.37 ± 3.96	7.9	1.96
	2470	2320.26 ± 10.40	0.5	-6.06	2296.49 ± 31.09	1.4	7.02
Meranzin	0.836	0.78 ± 0.06	7.2	-6.18	0.76 ± 0.05	6.5	-9.27
	16.72	15.27 ± 0.72	4.7	-8.66	15.25 ± 1.00	6.6	-8.80
	836	829.63 ± 7.05	0.9	-0.76	823.28 ± 11.34	1.4	-1.52
Nobiletin	0.4424	0.41 ± 0.03	7.9	-4.01	0.42 ± 0.05	10	0.29
	8.44	9.26 ± 0.29	3.2	9.68	9.28 ± 0.44	4.8	9.94
	422.4	410.48 ± 26.76	6.5	-2.82	376.29 ± 32.40	8.6	-10.92

### Extraction Recovery and Matrix Effect

The extraction recovery and matrix effect were summarized in **Table 4**. The recoveries were between 82.33 to 89.92%, and the matrix effects of the five compounds were in the range of 94.36–101.65%. These data indicate that the UPLC-MS/MS method provides suitable, reproducible, and reliable data and can be used as an analytical tool in rat plasma.

**Table 4:** Extraction recovery and matrix effects of the analytes in rat plasma (n = 6).

Components	Concentration (ng/mL)	Extraction recovery (%)		Matrix effect (%)	
		Mean ± SD	RSD(%)	Mean ± SD	RSD(%)
Meranzin hydrate	7.76	83.44 ± 5.37	6.4	100.28 ± 6.14	6.1
	77.6	87.89 ± 6.33	7.2	100.94 ± 4.85	4.8
	776	88.13 ± 3.40	3.5	97.88 ± 4.45	4.5
Naringenin	5.892	82.33 ± 1.58	1.9	94.36 ± 4.85	5.1
	58.92	86.12 ± 4.02	4.7	98.34 ± 6.36	6.5
	589.2	89.33 ± 1.88	2.1	100.31 ± 4.86	4.8
Hesperetin	4.94	87.79 ± 4.51	5.1	95.95 ± 7.08	7.4
	49.4	89.15 ± 6.10	6.8	99.51 ± 4.62	4.6
	494	88.93 ± 3.17	3.6	98.64 ± 1.65	1.7
Meranzin	1.672	82.97 ± 5.88	7.1	101.08 ± 4.97	4.9
	16.72	88.50 ± 4.46	5.0	96.90 ± 5.39	5.6
	167.2	88.04 ± 3.40	3.9	98.84 ± 7.72	7.8
Nobiletin	0.8448	83.63 ± 0.06	7.7	101.65 ± 6.80	6.7
	8.44	89.92 ± 1.78	2.0	100.17 ± 6.86	6.9
	84.48	89.85 ± 2.20	2.4	99.16 ± 6.83	6.9

### Stability

The stability data under different experimental conditions are summarized in **Table 5**. The results showed all RSD% were less than 8.8% and RE% were in the range from -13.50 to 14.81%, which suggested that rat plasma samples containing 5 analytes were stable under routine laboratory conditions and no additional procedures were necessary to stabilize the sample for pharmacokinetic studies.

**Table 5:** Stability of the 5 analytes in rat plasma (n = 6).

Components	Concentration (ng/mL)	Short-term Stability		Free–thaw Stability		Long-term Stability	
		RSD (%)	RE (%)	RSD (%)	RE (%)	RSD (%)	RE (%)
Meranzin hydrate	3.88	7.2	4.71	4.6	5.77	5.9	7.62
	77.6	1.9	1.55	3.8	0.71	2.2	3.19
	3880	0.5	7.43	1.5	7.63	2.6	6.85
Naringenin	2.946	2.8	-3.02	0.3	-4.36	7.6	-0.84
	58.92	1.2	14.81	2.8	8.14	1.2	12.09
	2946	3.9	-13.49	3.5	-10.94	4.2	-9.76
Hesperetin	2.47	7.6	4.10	10	7.78	4.7	5.03
	49.4	5.6	-9.20	5.0	-10.70	4.3	-8.58
	2470	8.7	-13.50	8.0	-12.68	5.3	-7.81
Meranzin	0.836	2.7	-6.25	1.6	-9.71	4.9	-10.98
	16.72	13	-8.40	1.6	11.07	8.8	-13.52
	836	2.1	-2.81	2.6	-6.01	5.9	-7.37
Nobiletin	0.4224	1.1	7.01	8.2	2.21	4.9	5.83
	8.44	4.3	7.89	2.5	10.46	3.9	6.71
	422.4	0.3	0.80	0.3	-1.72	1.7	-3.36

### Pharmacokinetics

**Figure 3:**
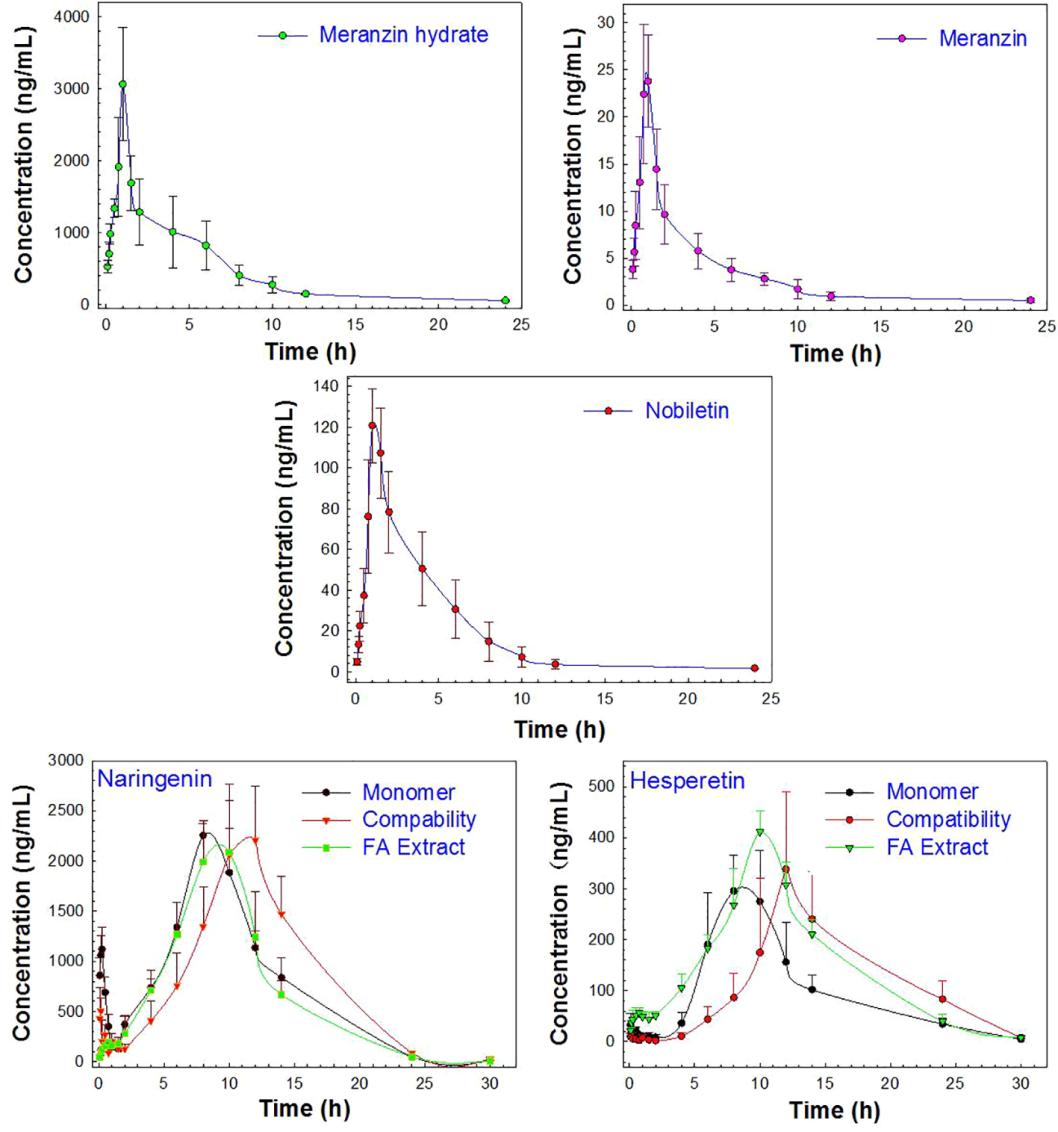
The profiles of the mean plasma concentration over time after oral administration of the monomer (NA or NHE), the compatibility (NA-NHE), and FA extract (FA) form (mean ± SD, n = 6).

**Figure 4:**
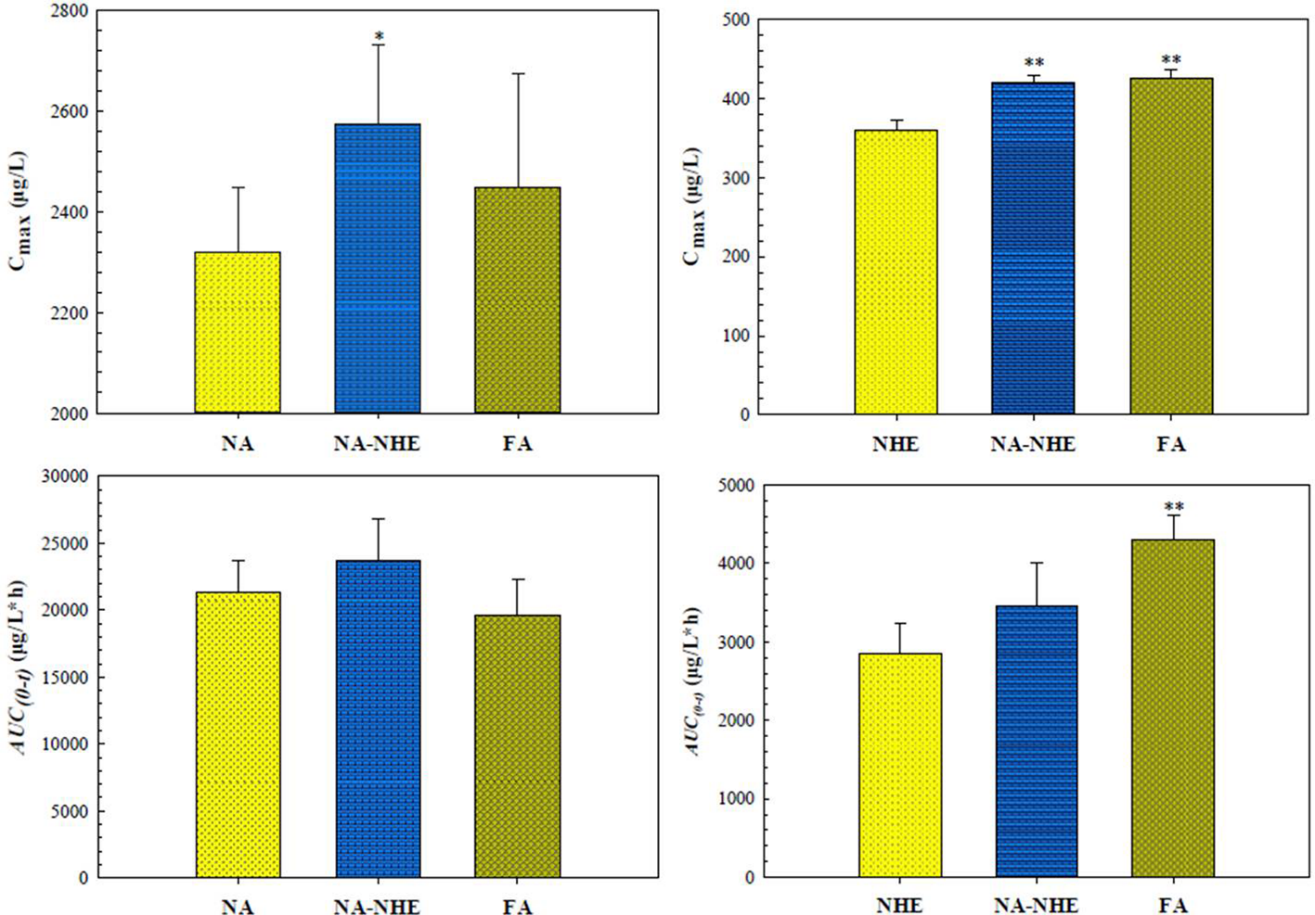
Comparison of C_max_ and AUC_(0-t)_ of naringin (NA) and neohesperidin (NHE) after oral administration of monomer (NA or NHE), the compatibility (NA-NHE), and FA extract (FA) form. (mean ± SD, n = 6). *P < 0.05, **P < 0. 01 for NA-NHE group & FA extract group *vs* monomer group.

The proposed UPLC-MS/MS method was applied to a multi-component pharmacokinetic study of meranzin hydrate, naringenin, hesperetin, meranzin, and nobiletin in SD rats after oral administration of the extract, NA, NHE, and NA-NHE. The plasma concentration-time profiles of the five components were shown in **Figure 3**, and the corresponding pharmacokinetic parameters were shown in **Tables 6** and **7**. The physiological disposition conformed to a two-compartment model of the 5 analytes in rats fitted by the DAS 3.1 software. The results suggested that there were significant differences in pharmacokinetic parameters of naringenin and hesperetin between different drug forms, including AUC_(0–t)_, AUC_(0–∞)_, C_max_, t_max_, t_1/2z_, and CL_Z/F_. The main differences including remarkable increases in the values of AUC_(0–∞)_, AUC_(0–t)_ and C_max_, obvious decrease of CL_Z/F_, and longer t_max_ and t_1/2z_. The comparisons of C_max_ and AUC_(0–t)_ of different groups were shown in **Figure 4**.

**Table 6:** Pharmacokinetic parameters of the 5 compounds in male SD rats after oral administration of FA extract (mean ± SD, n = 6).

Parameter	Components				
	Meranzin hydrate	Naringenin	Hesperetin	Meranzin	Nobiletin
C_max_ (µg/L)	3356.68 ± 91.03	2448.08 ± 225.62	424.89 ± 11.13	27.74 ± 2.85	130.28 ± 4.78
T_max_ (h)	0.96 ± 0.10	9.33 ± 1.03	9.67 ± 0.82	0.92 ± 0.13	1.17 ± 0.26
t_1/2z_ (h)	6.50 ± 1.20	2.56 ± 0.17	3.40 ± 0.46	6.52 ± 0.63	2.10 ± 0.37
AUC_(0-t)_ (µg/L*h)	11091.22 ± 2824.56	19617.11 ± 2623.90	4295.33 ± 314.81	76.90 ± 14.69	472.64 ± 118.70
AUC_(0-∞)_ (µg/L*h)	11620.50 ± 2763.67	19651.18 ± 2634.06	4337.25 ± 325.91	80.41 ± 15.49	480.11 ± 116.51
MRT_(0-t)_ (h)	5.17 ± 0.34	9.71 ± 0.56	11.42 ± 0.37	4.82 ± 0.76	4.23 ± 0.50
MRT_(0-∞)_ (h)	6.57 ± 0.95	9.75 ± 0.56	11.65 ± 0.42	6.39 ± 2.22	4.80 ± 0.99
V_z/F_ (L/kg)	9.36 ± 3.56	2.06 ± 0.29	12.24 ± 1.67	476.8 ± 70.2	150.97 ± 46.98
CLz/F (L/h/kg)	0.97 ± 0.23	0.56 ± 0.08	2.50 ± 0.18	138.66 ± 27.24	23.60 ± 5.67

**Table 7:** Pharmacokinetic parameters of naringin (NA) and neohesperidin (NHE) after oral administration of monomer, the compatibility of NA-NHE, and FA extract form (mean ± SD, n = 6).

Parameter	Groups of NA			Groups of NHE		
	NA	NA-NHE	FA	NHE	NA-NHE	FA
C_max_ (µg/L)	2317.25 ± 130.51	2573.79 ± 158.38*	2448.08 ± 225.62	358.85 ± 14.09	419.73 ± 9.88**	424.89 ± 11.13**
T_max_ (h)	8.67 ± 1.03	11.33 ± 1.03	9.33 ± 1.03	9.00 ± 1.10	12.00 ± 1.27	9.67 ± 0.82
t_1/2z_ (h)	2.70 ± 0.23	2.80 ± 0.35	2.56 ± 0.17	4.35 ± 0.81	3.13 ± 0.29	3.40 ± 0.46
AUC_(0-t)_ (µg/L*h)	21336.35 ± 2347.74	23670.00 ± 3087.55	19617.11 ± 2623.90	2848.13 ± 395.44	3448.04 ± 552.57	4295.33 ± 314.81**
AUC_(0-∞)_ (ug/L*h)	21386.91 ± 2368.44	23776.17 ± 3078.88	19651.18 ± 2634.06	2903.91 ± 396.58	3488.92 ± 571.92	4337.25 ± 325.91
MRT_(0-t)_ (h)	9.59 ± 0.29	11.48 ± 0.51	9.71 ± 0.56	11.29 ± 0.93	14.60 ± 1.30	11.42 ± 0.37
MRT_(0-∞)_ (h)	9.65 ± 0.30	11.58 ± 0.52	9.75 ± 0.56	11.88 ± 1.04	14.81 ± 1.40	11.65 ± 0.42
V_z/F_ (L/kg)	0.34 ± 0.04	0.32 ± 0.06	2.06 ± 0.29	3.96 ± 1.66	2.38 ± 0.31	12.24 ± 1.67
CLz/F (L/h/kg)	0.09 ± 0.01	0.08 ± 0.01	0.56 ± 0.08	0.63 ± 0.09	0.53 ± 0.10	2.50 ± 0.18

*P < 0.05, **P < 0. 01 for NA-NHE group & FA extract group vs monomer group.

## Discussion

UPLC-MS/MS is a powerful analytical tool for the trace compounds in biosamples with high selectivity and sensitivity (Yuan et al., 2017). Various influencing factors were investigated carefully including separation and detection conditions in this work, and the optimal analytical parameters were obtained for the studied analytes. In addition, the mass spectrometer can be switched freely between ESI (Electro-spray Ionization) +/- detection modes in a single injection, and it ensures that each analyte can get the best response. Therefore, the developed method achieved higher sensitivity than the literatures did with lower LODs (see **Table 2**).

A study (Wang et al., 2018) has reported that flavone glycosides (naringin, hesperidin, and neohesperidin) could not been detected in the rat plasma even after orally administering 30 g/kg FA decoction, which might be associated with the hydrolysis caused by microbial bacterial from the gastrointestinal tract (Hsiu et al., 2002). Naringin can be quickly absorbed, and metabolized into its aglycone and naringenin glucuronide. Hesperidin was also quickly transferred to hesperetin due to the similar structure (Hu et al., 2015; Wang et al., 2018). In general, naringin and hesperidin are hydrolyzed in the gastrointestinal tract by the enzymes of intestinal bacteria followed by absorption and conjugation of their aglycones, and the main present forms are naringenin and hesperitin glucuronide/sulfatase conjugations and a small amount of the free aglycones in plasma (Xu et al., 2019). Therefore, the concentration of naringin and neohesperidin in mouse plasma were expressed respectively by the total (free + conjugated) concentration of naringenin and hesperitin which was determined by UPLC-MS/MS after hydrolysis with *β*-glucuronidase and sulfatase in this work.

As shown in **Figure 3**, the change trend in concentration-time profiles of naringenin and hesperetin are basically consistent with previous reports (Xu et al., 2010; Tong et al., 2012; Xu et al., 2019): both had double peaks. The double-peak phenomenon may be due to the enterohepatic circulation and drug reabsorption of naringin and neohesperidin in rats, which was also reported for other flavonoid glycosides from the extract of TCM (Ma et al., 2006; Lu et al., 2007; Zhang et al., 2012; Okada et al., 2017). In addition, these components can be detected in rat plasma within 5 min after dosing with a short T_max_ (less than 1 h), and which indicates that they be absorbed easily and rapidly after oral administration. The values of C_max_ and AUC of naringenin and hesperitin are relatively big, which indicates their bioavailability is not as low as reported in the literatures (Ma et al., 2006). The metabolic behaviors of the flavonoid glycosides might also be explained by the enterohepatic circulation or multiple site absorption (Wang et al., 2016). Meanwhile, the double-peak phenomenon and the so-called low bioavailability might also suggest that the reabsorption of the metabolites secreted in bile for FA should be more obvious.

Meranzin hydrate and meranzin, as the typical coumarins, were absorbed rapidly (T_max_, about 1 h) but eliminated slowly (t_1/2z_ exceeds 6.5 h). Nobiletin, a polymethoxy flavone, was also rapidly absorbed with T_max_ value of 1.17 ± 0.26 h and AUC_(0-t)_ value of 472.64 ± 118.70, which was consistent with the previous report (Onoue et al., 2013; Shen et al., 2019). The average t_1/2z_ of nobiletin was 2.10 ± 0.37 h in rats, suggested that nobiletin should be rapidly distributed and eliminated (Shen et al., 2019), which was partly different from the previous report, probably owing to the interactions among components in FA extract (Qiao et al., 2012; Zhang et al., 2018).

Statistical moment analysis (corresponding to non-compartmental model) and compartmental model analysis can be simultaneously performed in a run of batch analysis by DAS 3.1. In the current *in vivo* data analysis, the non-compartmental model has become the mainstream processing method and has been recommended by the drug review authorities in various countries (Yuan et al., 2017; Xu et al., 2019). In particular, the statistical moment analysis is more reliable for the drugs with double-peak phenomena. In this work, naringenin and hesperetin are found to have exactly these pharmacokinetic properties. So, the statistical moment parameters are shown in **Tables 6** and **7**. In the compartmental model analysis, various models and different statistical weights were compared in this work, and two-compartment parameters were found to be the optimal fitting values with the minimum AIC (Akaike's Information Criterion) and the best R (correlation coefficient) values (Yuan et al., 2017; Yang et al., 2019). The compartmental model parameters are shown in **Tables S1** and **S2**.

As mentioned above, the pharmacokinetic characteristics of naringenin and hespeietin are closely related with enterohepatic circulation or multiple site absorption/reabsorption, which indicates their pharmacokinetics may be affected by the forms in which they exist. Therefore, we design the following four groups to investigate the effects: pure monomer (naringenin and hesperitin), compatibility (NA-NHE), and FA extract group. The results demonstrated that there were significant differences in pharmacokinetic parameters between different drug forms, including AUC_(0–t)_, AUC_(0–∞)_, C_max_, t_max_, t_1/2z_, and CL_Z/F_. The main differences were observed between the compatibility group and the other two groups: ① remarkable increases in the values of AUC_(0–∞)_, AUC_(0–t)_ and C_max_; ② obvious decrease of CL_Z/F_; and ③ longer t_max_ and t_1/2z_. The comparisons of C_max_ and AUC_(0–t)_ between different groups were shown in **Figure 4**. The results suggest that compatibility can promote mutual absorption and affect the metabolic behaviors. Drug-drug interactions often occur, and the detailed effect of compatibility on the pharmacokinetics needs further study.

## Conclusion

The proposed UPLC-MS/MS method was suitable for the accurate determination of meranzin hydrate, naringenin, hesperetin, meranzin, and nobiletin in rat plasma with high selectivity and sensitivity. The analytical method has been successfully used for the pharmacokinetic studies following oral administration of monomer, drugs in compatibility, and FA extract. The five components in FA can rapidly be absorbed. Drug compatibility can promote mutual absorption and affect the metabolic behaviors.

## Data Availability Statement

All datasets generated for this study are included in the article/**Supplementary Material**.

## Ethics Statement

The animal study was reviewed and approved by The Experimental Animal Ethic Committee of Jiangxi University of Traditional Chinese Medicine.

## Author Contributions

JY, FW, and WY conceived and designed the experiments. FW, XL, RQ, MZha, MZho, and HC performed the experiments. FW, JY, and WY analyzed the data. JY and FW wrote the paper. All authors contributed to the article and approved the submitted version.

## Funding

This work was financially supported by the National Natural Science Foundation of China (No. 81860698 and 81760680) and the Double First-class Discipline of Jiangxi Province (No. JXSYLXK-ZHYAO064).

## Conflict of Interest

The authors declare that the research was conducted in the absence of any commercial or financial relationships that could be construed as a potential conflict of interest.
