# Supplementary material for: Multi-Component Comparative Pharmacokinetics in Rats After Oral Administration of Fructus aurantii Extract, Naringin, Neohesperidin, and Naringin-Neohesperidin
Source: Front Pharmacol. 2020 Jun 19;11:933. doi: 10.3389/fphar.2020.00933 (PMC7319089; doi:10.3389/fphar.2020.00933)
Supplement: Supplementary file 1 [file Image_1.pdf]

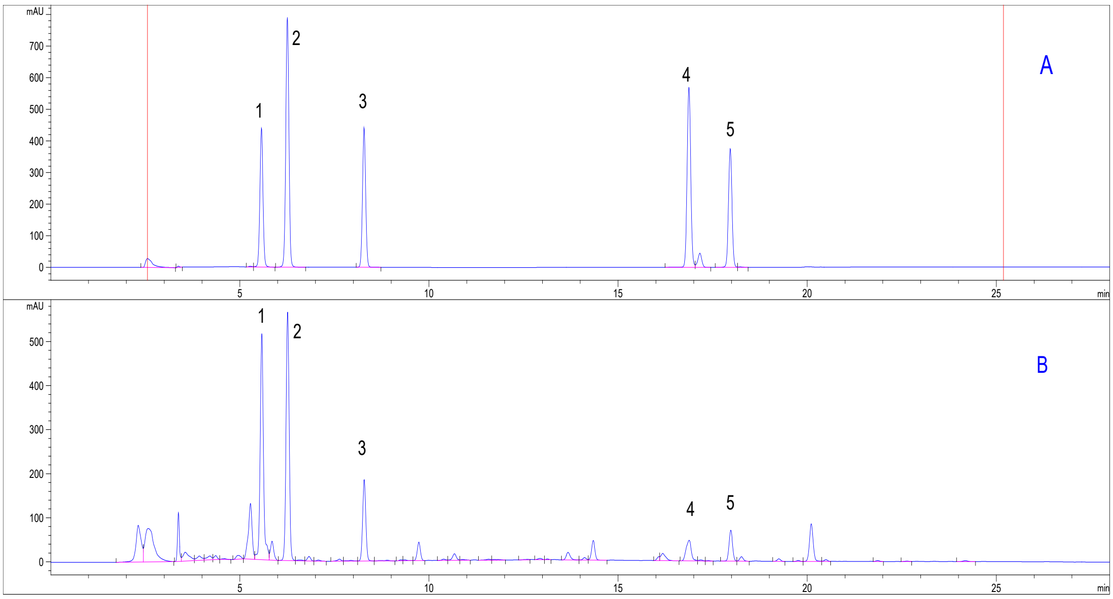

Peak No.	Components	Content (mg/g raw herb) (mean ± SD)
1	Naringin	171.6 ± 1.3
2	Neohesperidin	167.5 ± 1.1
3	Meranzin hydrate	1.98 ± 0.18
4	Meranzin	0.94 ± 0.08
5	Nobiletin	1.70 ± 0.10

Fig.S1 Typical chromatograms of samples and the contents of the five components. A. The mixed standard; B. the FA extract
