# Supplementary material for: Multi-Component Comparative Pharmacokinetics in Rats After Oral Administration of Fructus aurantii Extract, Naringin, Neohesperidin, and Naringin-Neohesperidin
Source: Front Pharmacol. 2020 Jun 19;11:933. doi: 10.3389/fphar.2020.00933 (PMC7319089; doi:10.3389/fphar.2020.00933)
Supplement: Supplementary file 2 [file Table_1.docx]

**Table S1 |** Compartmental model parameters of the 5 compounds in male SD rats after oral administration of FA extract (mean ± SD, n = 6).

| **Parameter** | **Components** | | | | |
| --- | --- | --- | --- | --- | --- |
|  | **Meranzin hydrate** | **Naringenin** | **Hesperetin** | **Meranzin** | **Nobiletin** |
| t_1/2α_（h） | 6.61±0.86 | 2.63 ± 0.39 | 3.47 ± 0.51 | 6.39 ± 0.11 | 4.40 ± 0.34 |
| t_1/2β（_h） | 6.91±1.05 | 2.65 ± 0.18 | 3.86 ± 0.32 | 6.48 ± 0.38 | 4.49 ± 0.57 |
| V_1/F_（L/kg） | 4.30 ± 1.14 | 0.82 ± 0.06 | 7.17 ± 1.40 | 5.91 ± 1.12 | 6.80 ± 1.24 |
| CL/F（L/h/kg） | 0.45 ± 0.18 | 0.83 ± 0.17 | 4.09 ± 1.10 | 72.15 ± 64.90 | 8.05 ± 3.52 |
| AUC_(0-t)_（µg/L*h） | 12106.50 ± 2760.12 | 20730.29 ± 3006.69 | 4345.39± 704.69 | 84.38±22.49 | 472.19±62.65 |
| AUC_(0-∞)_（µg/L*h） | 12260.64 ± 2594.67 | 20850.93 ± 3012.65 | 4421.59± 723.98 | 87.80±22.89 | 473.21±63.09 |
| Ka（1/h） | 2.05 ± 0.46 | 1.94 ± 0.37 | 3.17 ± 0.93 | 2.18 ± 0.97 | 0.69 ± 0.25 |
| t1/2_(Ka)_（h） | 0.32 ± 0.06 | 0.05 ± 0.01 | 0.26 ± 0.07 | 0.35 ± 0.10 | 1.46 ± 0.32 |
