# Supplementary material for: Multi-Component Comparative Pharmacokinetics in Rats After Oral Administration of Fructus aurantii Extract, Naringin, Neohesperidin, and Naringin-Neohesperidin
Source: Front Pharmacol. 2020 Jun 19;11:933. doi: 10.3389/fphar.2020.00933 (PMC7319089; doi:10.3389/fphar.2020.00933)
Supplement: Supplementary file 3 [file Table_2.docx]

**Table S2** | Two-compartment parameters of naringin (NA) and neohesperidin (NHE) after oral administration of monomer, the compatibility of NA-NHE, and FA extract form (mean ± SD, n = 6).

| **Parameter** | **Groups of NA** | | | **Groups of NHE** | | |
| --- | --- | --- | --- | --- | --- | --- |
|  | **NA** | **NA-NHE** | **FA** | **NHE** | **NA-NHE** | **FA** |
| t_1/2α_（h） | 2.67 ± 0.22 | 2.77 ± 0.20 | 2.63 ± 0.39 | 4.39 ± 0.47 | 3.22 ± 0.42 | 3.47 ± 0.51 |
| t_1/2β_（h） | 2.84 ± 0.16 | 2.87 ± 0.31 | 2.65 ± 0.18 | 4.61 ± 0.48 | 3.43 ± 0.55 | 3.86 ± 0.32 |
| V_1/F_（L/kg） | 10.56 ± 1.87 | 3.29 ± 0.61 | 0.82 ± 0.06 | 4.58 ± 1.18 | 4.37 ± 1.16 | 7.17 ± 1.40 |
| CL/F（L/h/kg） | 0.863 ± 0.17 | 0.72 ± 0.16 | 0.83 ± 0.17 | 5.49 ± 0.73 | 3.63 ± 0.80 | 4.09 ± 1.10 |
| AUC_(0-t)_（µg/L*h） | 21995.33 ±1182.34 | 23554.39 ± 1766.13 | 20730.29 ± 3006.69 | 2932.05 ± 625.03 | 3475.14 ± 714.19 | 4345.39± 704.69 |
| AUC_(0-∞)_（µg/L*h） | 22732.57 ± 1704.17 | 23807.25 ± 1842.63 | 20850.93 ± 3012.65 | 2988.05 ± 621.39 | 3557.64 ± 726.84 | 4421.59± 723.98 |
| Ka（1/h） | 5.29 ± 0.83 | 4.14 ± 1.02 | 1.94 ± 0.38 | 1.64 ± 0.35 | 4.40 ± 0.83 | 3.17 ± 0.93 |
| t_1/2(Ka)_（h） | 0.06 ± 0.01 | 0.077 ± 0.01 | 0.049 ± 0.01 | 0.96 ± 0.08 | 0.57 ± 0.11 | 0.26 ± 0.07 |
